# Evaluation of Drought Implications on Ecosystem Services: Freshwater Provisioning and Food Provisioning in the Upper Mississippi River Basin

**DOI:** 10.3390/ijerph14050496

**Published:** 2017-05-08

**Authors:** Ping Li, Nina Omani, Indrajeet Chaubey, Xiaomei Wei

**Affiliations:** 1 College of Water Resources and Architectural Engineering, Northwest A&F University, 23 Weihui Road, Yangling 712100, China; weixiaomei@nwsuaf.edu.cn; 2 Department of Earth, Atmospheric, and Planetary Sciences, Purdue University, 550 Stadium Mall Drive, West Lafayette, IN 47907, USA; ninaomani@gmail.com (N.O.); ichaubey@purdue.edu (I.C.); 3 Department of Agriculture and Biological Engineering, Purdue University, 225 South University Street, West Lafayette, IN 47907, USA

**Keywords:** drought, ecosystem services, Upper Mississippi River Basin, Soil and Water Assessment Tool (SWAT)

## Abstract

Drought is one of the most widespread extreme climate events with a potential to alter freshwater availability and related ecosystem services. Given the interconnectedness between freshwater availability and many ecosystem services, including food provisioning, it is important to evaluate the drought implications on freshwater provisioning and food provisioning services. Studies about drought implications on streamflow, nutrient loads, and crop yields have been increased and these variables are all process-based model outputs that could represent ecosystem functions that contribute to the ecosystem services. However, few studies evaluate drought effects on ecosystem services such as freshwater and food provisioning and quantify these services using an index-based ecosystem service approach. In this study, the drought implications on freshwater and food provisioning services were evaluated for 14 four-digit HUC (Hydrological Unit Codes) subbasins in the Upper Mississippi River Basin (UMRB), using three drought indices: standardized precipitation index (*SPI*), standardized soil water content index (*SSWI*), and standardized streamflow index (*SSI*). The results showed that the seasonal freshwater provisioning was highly affected by the precipitation deficits and/or surpluses in summer and autumn. A greater importance of hydrological drought than meteorological drought implications on freshwater provisioning was evident for the majority of the subbasins, as evidenced by higher correlations between freshwater provisioning and *SSI*12 than *SPI*12. Food provisioning was substantially affected by the precipitation and soil water deficits during summer and early autumn, with relatively less effect observed in winter. A greater importance of agricultural drought effects on food provisioning was evident for most of the subbasins during crop reproductive stages. Results from this study may provide insights to help make effective land management decisions in responding to extreme climate conditions in order to protect and restore freshwater provisioning and food provisioning services in the UMRB.

## 1. Introduction

Ecosystem services have become an important component in environmental management and policy making. The concept of ecosystem services has been broadly defined by the Millennium Ecosystem Assessment (MEA, 2005) as the benefits humans obtain from ecosystems, and includes provisioning, regulating, cultural, and supporting services [1]. Freshwater availability is an important component of human well-being and a thriving economy. The supply of freshwater is a critical provisioning service describing the ecosystem modification of water used for multiple purposes, such as drinking, irrigation, hydropower production, recreation, fisheries, etc. [2,3]. In addition to freshwater, food supply is another important provisioning service, and is tightly linked with freshwater provisioning [4].

Climate change and variability increase intensity and frequency of drought events with cascading impacts on freshwater and food provisioning. Drought usually onsets with a deficiency of precipitation over an extended period of time [5]. The water deficit during droughts propagates through the hydrological cycle, reducing the streamflow, lake levels as well as groundwater levels, and is often termed as hydrological drought. Stream water quality may deteriorate due to prolonged low-flow conditions and high water temperatures that occur during drought periods [6,7,8]. Droughts impacting water availability and quality can potentially have considerable influence on freshwater provisioning. In addition to freshwater, drought may limit the plant growth and crop yield because of precipitation and soil moisture deficits, with a potential to negatively impact food provisioning services. Given the importance of freshwater and food provisioning in sustaining food security and economic development, evaluation of drought implications on these ecosystem services is necessary.

Studies related to the concept and valuation of ecosystem services have considerably increased in recent years. The importance of incorporating ecosystem services into land-use/management decisions is now generally recognized. However, studies related to quantitative estimates of ecosystem services are still limited [3,4,9,10]. Even relatively fewer studies have been conducted to quantitatively assess the ecosystem services in relation to extreme climate events such as drought [10,11,12]. Bangash et al. [11] and Terrado et al. [12] evaluated changes in delivery of freshwater provision and erosion control services in extreme dry and wet years in a Mediterranean river basin by using Integrated Valuation of Ecosystem Services and Tradeoffs (InVEST)—an ecosystem service tool. The authors reported that drinking water provisioning services decreased by 3–49% while erosion control reduced by 5–43% under future scenarios with an increased frequency of floods and droughts. The drinking water and hydropower production were almost 100% reduced compared to normal years, while water purification service provided higher benefits in dry years [12]. Studies about drought implications on hydrology and water quality have substantially increased in the past 20 years [7,8,13,14]. Van Vliet and Zwolsman [8] evaluated the summer droughts impacts on water quality of Meuse River and indicated that drought adversely affected water quality with respect to water temperature, eutrophication, and some heavy metals and metalloids, while positive effects on nitrate were observed during droughts. Similarly, several studies about drought implications on crop yields have been published recently [15,16,17,18]. For instance, Wang et al. [18] estimated the effects of climate variability on crop yield and concluded that the total yield was reduced by 8.1–17.5% due to drought stress at their four study sites in the Midwest USA. However, these studies have primarily focused on drought implications on variables such as streamflow, nutrient loads, and crop yields. These variables are all process-based model outputs that could represent ecosystem functions that contribute to the ecosystem services [4]. Studies evaluating drought effects on ecosystem services such as freshwater and food provisioning and quantifying freshwater and food provisioning by use of an ecosystem service-based approach are very sparse [4,11,12]. For instance, Logsdon and Chaubey [4] proposed an index-based ecosystem service approach to quantify five provisional and regulatory ecosystem services. They demonstrated the effectiveness of their methods in a mixed land use watershed in Indiana, which could be a building block to quantify the full suite of ecosystem services.

The Upper Mississippi River Basin (UMRB) is a significant agricultural production area as well as a major contributor of nutrients to the Gulf of Mexico [19,20,21]. Given the high sensitivity of hydrology and ecosystem structure to climate changes, we selected UMRB as the study site. The main objective of this study was to assess the drought implications on freshwater provisioning and food provisioning services in the UMRB. To achieve this objective, Soil and Water Assessment Tool (SWAT)—a process-based model—was set up to capture the ecosystem functions as inputs for quantifying freshwater provisioning and food provisioning services for 14 four-digit HUC (Hydrological Unit Codes, HUC4) subbasins in the UMRB. Three representative drought indices (standardized precipitation index (*SPI*), standardized soil water content index (*SSWI*), and standardized streamflow index (*SSI*)) were calculated for drought characteristic analysis and for evaluating drought implications on freshwater and food provisioning services in the UMRB.

## 2. Materials and Methods

### 2.1. Study Area

**Figure 1:**
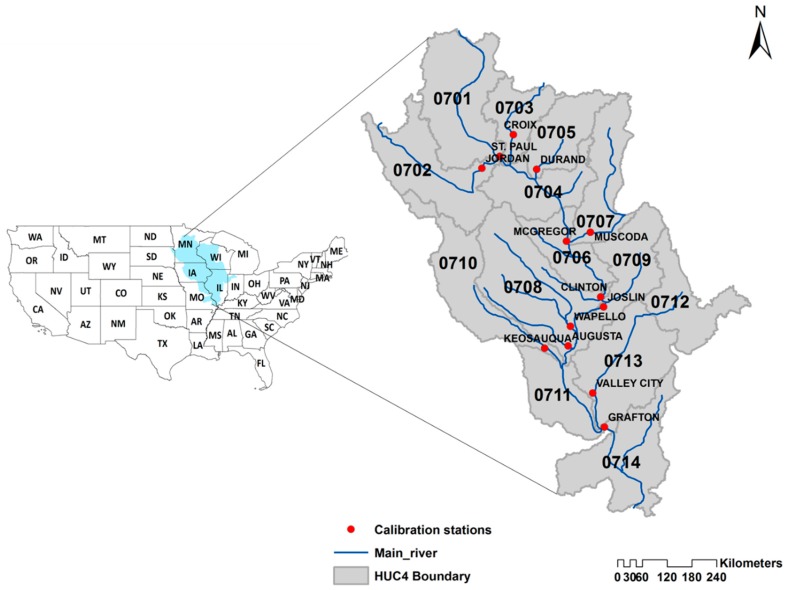
Location of the Upper Mississippi River Basin (UMRB) displaying four-digit Hydrological Unit Coded (HUC4) subbasins and calibrated streamflow gauge stations.

The UMRB is the headwater basin of the Mississippi River, which is the largest river in North America (Figure 1). It stretches 2100 km from Lake Itasca in Minnesota to the confluence with the Ohio River just north of Cairo in Illinois, USA. The basin covers approximately 492,000 km^2^ and includes large sections of Minnesota, Wisconsin, Iowa, Illinois, and Missouri. The UMRB consists of 14 HUC4 subbasins and 131 HUC8 watersheds (Figure 1; Table 1). Climate conditions in the basin vary considerably because of the north-south flow across the temperate zone of North America. The average annual air temperature in the basin ranges from 3 °C in the north to 15 °C in the south. The average annual precipitation in the basin is approximately 900 mm, ranging from 600 mm/year in the north to 1220 mm/year in the south [22,23]. The UMRB is an agriculturally-dominant river basin and over 60% of land use is cropland or pasture, with the major cash crops being corn and soybeans. The remainder of the land uses mainly are forests, wetlands, haylands, lakes, and urban areas [23]. The basin is also a key contributor to the hypoxia in the Gulf of Mexico. Although it covers only 15% of drainage area of the Mississippi/Atchafalaya River Basin(MARB), about 43% of the nitrate load and 26% of the total phosphorus load from UMRB were delivered to the Gulf of Mexico during 2001–2005 [21,24]. Extensive anthropogenic activities such as conversion of the historic prairie and forest landscape to agriculture and the alteration of the rivers for navigation and flood control have adversely impacted the ecological integrity and ecosystem structures in the basin [23,25].

### 2.2. SWAT Model Set-Up and Calibration

SWAT is a conceptual, distributed hydrological model developed by United States Department of Agricultural [26,27]. The model was developed to evaluate various land use/management effects on hydrology, water quality, and crop production [28,29]. A multitude of outputs at various spatiotemporal scales predicted by SWAT can be used for evaluation of ecosystem services. Based on global applicability, SWAT was selected in this study to quantify the freshwater provisioning and food provisioning services in the UMRB [4,30].

Spatially, SWAT divides a basin into subbasins and then further into smaller homogeneous units called hydrologic response units (HRUs), which represent unique land-use, soil, and slope combinations. As a physically-based hydrological model, SWAT requires a considerable amount of input data including weather, hydrography, topography, land use/cover data, soil data as well as management practices to derive parameters and simulate crop growth and hydrological processes [28]. In this study, the predefined watershed and stream dataset and a 90-m resolution digital elevation model (DEM) were used for topographic parameter estimation. A total of 157 HUC8 subbasins were defined by the model for the UMRB. The land use layer of the model was created by using the U.S. Department of Agriculture (USDA)-National Agricultural Statistics Service (NASS) Cropland Data Layer (CDL) datasets. Nine years of CDL datasets (2006–2014) were overlaid to generate crop rotations used in the region. The USDA Natural Resources Conservation Services (USDA-NRCS) State Soil Geographic (STATSGO) soil map at 1:250,000 scale was used as soil input. Three slope classes (0–2%, 2–6%, >6%) were used for HRU classification. A total of 6686 HRUs were delineated by using the threshold values of 5% for land use, 10% for soil, and 5% for slope, respectively. Historic daily precipitation and maximum and minimum temperature data (1 January 1985 to 31 December 2014) recorded at 440 weather stations were derived from the National Climatic Data Center (NCDC) and used in this study.

The crop planting/harvesting dates and tillage practices were estimated based on county values from NASS data [31]. Fertilizer application rates were estimated based on Tri-State recommendations [24,32,33]. The tile drain areas were estimated in corn/soybean areas with less than 2% slope and poorly to very poorly drained soils from the STATSGO soil database.

The SWAT model calibration period was from 1995 to 2005 and the validation period was from 2006 to 2014. The model was calibrated using SWAT-CUP (Calibration and Uncertainty Procedures, [34]). SWAT-CUP is a software package that provides a generic interface to easily connect calibration/uncertainty or sensitivity program with SWAT [34]. It provides five different optimization programs for model calibration and includes functionalities for validation and sensitivity analysis. In this study, we used the Sequential Uncertainty Fitting (SUFI-2) program for model calibration, since this method is quite efficient for large-scale model simulations. The SUFI-2 requires fewer simulations to complete a calibration process and has been successfully applied for regional auto-calibration of relatively large areas [24,32]. A choice of 10 objective functions for calibration such as coefficient of determination (r^2^), Nash-Sutcliffe efficiency (NSE), and mean square error (MSE) are available to a user. In this study, we used the NSE as the main objective function for flow, sediment, as well as nutrient load calibration. Streamflow was calibrated using monthly data obtained from 13 US Geological Survey (USGS) gauge stations located closely to the outlet of subbasins (Figure 1). The in-stream sediment, total nitrogen (*TN*), and total phosphorus (*TP*) yield from six USGS sites were calibrated using the monthly time-series developed with the use of USGS’S LOAD ESTimator (LOADEST) program, as only sparser concentration samples of sediment and nutrients data were available [35]. Two major crop yields (corn and soybean) were also calibrated and validated for the period from 1995–2014. SWAT simulated crop yields were compared with the area weighted mean annual estimated yield reported by National Agricultural Statistics Service (NASS) county-level crop data. As the NASS data are reported by county and many counties have missing data, the yield data were aggregated to HUC4 subbasins based on area weighted method and then the results were compared with the SWAT simulated yield for the same HUC4 subbasins.

### 2.3. Drought Indices

Many drought indices have been proposed for characterizing droughts. Generally, drought is classified into three physical types: meteorological drought caused by precipitation deficits, agricultural drought resulting from soil moisture deficits, and hydrological drought due to the shortage of streamflow, inflows to reservoirs, lakes, and ponds, etc. [36,37]. In this study, we selected three indices: *SPI* for meteorological drought, *SSWI* for agricultural, and *SSI* for hydrological drought, respectively, to analyze drought implications on freshwater and food provisioning services.

#### 2.3.1. Standardized Precipitation Index (*SPI*)

The *SPI* (McKee et al. [38]) is one of the most popularly used meteorological drought indices. It is a flexible index that can be calculated at multiple timescales ([36,39]. The precipitation data is first fitted to a gamma distribution. The cumulative probability gamma function is then transformed into a standard normal distribution with a mean of zero and standard deviation of one. The *SPI* is the “z-score” in the standard normal distribution and represents the number of standard deviations its cumulative precipitation deficit/surplus deviates from the normalized mean at which a drought/wet event occurs. The drought is defined to occur when a Z value less than zero is consistently observed and reaches a value ≤−1 [37,38]. A drought event was categorized as an extreme drought if *SPI* was ≤−2, a severe drought if *SPI* was between −1.99 and −1.5, and a moderate drought if *SPI* was between −1.49 and −1. Extreme wet was classified if *SPI* was ≥2, severe wet was classified if *SPI* was between 1.5 and 1.99, and moderate wet was defined if *SPI* was between 1 and 1.49. The detailed description of *SPI* and the computer programs for calculating *SPI* are available at the National Drought Mitigation Center web site (http://www.drought.unl.edu/monitor).

Given that the *SPI* can be calculated at multiple time scales, a 3-month *SPI* (*SPI*3) reflects the short- and medium-term moisture conditions that can be used as an indicator of short-term water supplies such as the soil moisture availability for crop production. The 12-month *SPI* (*SPI*12) reflects the long-term precipitation patterns typically used to monitor the long-term streamflow, and reservoir and groundwater levels [40]. In this study, *SPI*3 and *SPI*12 were calculated for each of the HUC4 subbasins using monthly observed precipitation data in order to analyze short- and long-term drought (*SPI*3, *SPI*12) implications on freshwater provisioning, and the effects of seasonal droughts (*SPI*3) on food provisioning.

#### 2.3.2. Standardized Streamflow Index (*SSI*) and Soil Water Content Index (*SWCI*)

The streamflow deficit was monitored using *SSI* in this study. The *SSI* is defined using the concept of *SPI*, which is based on the probability of streamflow at different time scales [41,42]. The calculation procedure of *SSI* is similar to *SPI*, and includes the following steps [36]: (1) the time series of streamflow during 1985 and 2014 for each of the HUC4 subbasins were obtained from SWAT model simulation; (2) the streamflows were fitted to probability distributions; (3) the cumulative probability of streamflow values were estimated using the fitted distributions; and (4) the cumulative probability was transformed into the z-value of a normal distribution with mean of zero and variance of one.

Soil moisture is an important indicator of drought for agriculture and other sectors [42]. The Standardized Soil Water Content Index (*SSWI*) was used to represent the agricultural drought. The soil water content time series obtained from model simulations were fitted to a lognormal distribution and applied to calculate the *SSWI* using the similar calculation procedure of *SPI* and *SSI* [36]. The *SSI* and *SSWI* were calculated at both 3-month and 12-month scales to represent short- and long-term hydrological and agricultural droughts, respectively.

### 2.4. Ecosystem Services Quantification

A set of ecosystem service indices developed by Logsdon and Chaubey [4] were employed to quantify freshwater provisioning and food provisioning services for 14 HUC4 subbasins in the UMRB. This method is flexible and can be applied based on either observation of hydrologic and agricultural processes or the model simulated outputs to examine the ecosystem services in agricultural areas. Here, we describe the method in brief; more detailed information can be obtained from Logsdon and Chaubey [4].

#### 2.4.1. Freshwater Provisioning Calculation

The freshwater provisioning indicates the quantity of freshwater that can be provided for multiple uses (drinking, fisheries, recreation, and survival conditions for aquatic lives) in the case that water conditions simultaneously meet the environmental flow requirements and water quality standards [43]. The freshwater provisioning (*FWP*) and freshwater provisioning index *(FWPI)* at both seasonal and yearly scales were calculated from 1985 to 2014 for 14 HUC4 subbasins based on SWAT outputs of daily streamflow and water quality concentrations of *TN*, *TP*, and *TSS*. The equations were as following:

(1) $$FWP_{t}=Q_{t}\;\times \;FWPI_{t}$$

(2) $$FWPI_{t}=\frac{MF_{t}/MF_{EF}}{\left(MF_{t}/MF_{EF}+qne_{t}/n_{t}\right)}\;\times \;\frac{WQI_{avg,t}}{\left(1+e_{t}/n_{t}\right)}$$

(3) $$WQI=\frac{\exp\left(W_{1}+W_{2}+\cdots +W_{n}\right)}{exp\left[\left(W_{1}\times C_{1}/C_{1std}\;\right)+\left(W_{2}\times C_{2}/C_{2std}\right)+\cdots +\left(W_{n}\times C_{n}/C_{nstd}\right)\right]}$$

where *FWP_t_* is the quantity of freshwater provision (m^3^); *Q_t_* is the total flow (m^3^); *FWPI_t_* is the freshwater provision index; *MF* is the mean flow (m^3^/s); *MF_EF_* is the long-term environmental flow requirements (m^3^/s); *qne_t_* is the number of times that flow is less than the long-term environmental flow requirements in time step; *n* is the number of units in time step (i.e., 365/366 if *FWP* is calculated for the year on a daily basis); *WQI_avg,t_* is the average water quality index; *e* is the number of times that the *WQI* is below one. *C*_1_, *C*_2_ *… C*_n_ are concentrations of water quality constituents in water bodies; *w*_1_, *w*_2_ *… w*_n_ are the weights for nutrients concentrations; a std subscript represents the water quality standard for different nutrients; a *t* subscript denotes the time step. Both annual and seasonal time step were utilized for *FWP* and *FWPI* calculation in this study. The daily streamflow and nutrients concentration data are required inputs. In addition to three variables (*WQI*, *qne_t_*, and *e_t_*) that were calculated daily, other variables (*FWP_t_*, *FWPI_t_*, *WQI_avg,t_*) in equations were calculated at annual and seasonal scales, respectively.

As recommend by Tennant (1976), 30% of average flow was required to sustain good aquatic ecosystem functioning. Additionally, 10% of average flow was needed to provide short-term survival conditions for most aquatic lives [44]. Consequently, the *MF_EF_* used in this study was 30% of average annual flow. Two seasonal environmental flow values (October–March and April–September) for *qne* calculation were determined as 10% of average seasonal flow [4,43,44]. According to State Nutrient Reduction Strategy documents and water quality criteria in different ecoregions proposed by the Environmental Protection Agency (EPA) [45], the water quality standards of *TN*, *TP*, and *TSS* adopted in this study were 3 mg/L, 0.1 mg/L, and 38 mg/L, respectively. Using this method, if the environmental flow requirements and/or water quality standards are met, the *FWPI* will be equal to one, and the *FWP* is equal to the total quantity of water provided, indicating good freshwater provisioning. Conversely, if the long-term environmental flow requirements and/or water quality standards are not met, the *FWPI* will be less than one, causing the *FWP* to be less than the total quantity of water provided. This indicates that the freshwater provisioning is reduced during this time period.

#### 2.4.2. Food Provisioning Calculation

The food provisioning was evaluated using the food provisioning quantity (*FP*) and food provisioning index (*FPI*). Both were calculated annually from 1985 to 2014 for each HUC4 subbasin using the SWAT output of annual crop yield. The detailed descriptions of equation can be obtained from Logsdon and Chaubey [4]. Since corn and soybean are major cash crops cultivated in the basin, these two crops were considered for *FP* calculation. The minimum yield used for corn and soybean was the 30-year area weighted county yield derived from NASS data [4].

### 2.5. Impacts of Drought on FWP and FP

The Pearson product moment correlation (r) values between annual *FWP* and time series of *SPI*12 for each month, as well as the r values between seasonal *FWP* and *SPI*3 for each month within 1985–2014, were calculated to analyze the short-term drought effects on seasonal freshwater provisioning and long-term drought implications on annual freshwater provisioning for each subbasin. The probability values (*p*-values), in the case that the hypothesis of no correlation is true, were used to test the statistical significance of correlation results. The *SPI* considers the precipitation as the climate factor and it reflects meteorological drought effects on *FWP*. When compared to precipitation, the *FWP* is more directly affected by flow and water quality concentrations. These two factors are reflected by hydrological drought. Thus, correlations between annual *FWP* and *SSI* at 12-month scale (*SSI*12) were also analyzed to evaluate the hydrological drought implications on *FWP*.

Similarly, the *SPI*3 was selected to perform a correlation analysis with annual *FP*, since *SPI*3 reflects the short- and medium-term soil water supplies which are important for crop production. The correlation coefficients between annual *FP* and *SSWI* at a 3-month scale (*SSWI*3) were also calculated to analyze the agricultural drought effects on *FP*.

## 3. Results

### 3.1. SWAT Model Calibration and Validation

**Figure 2:**
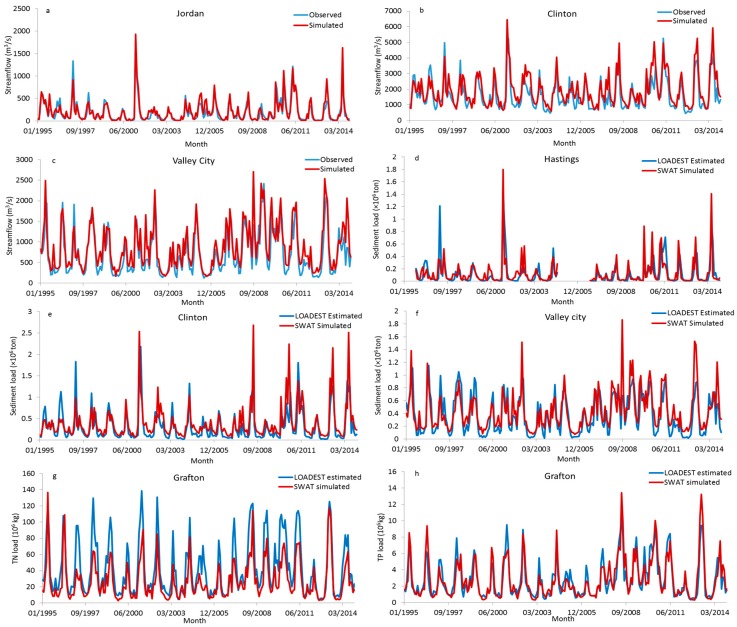
Monthly simulated versus observed streamflow, sediment, total nitrogen (*TN*), and total phosphorus (*TP*) loads for some stations in the UMRB.

The monthly streamflow calibration and validation results indicate a satisfactory model performance for hydrologic prediction [46]. The r^2^ values in calibration and validation periods were ≥0.7 for almost all stations, and ranged from 0.66 to 0.85 in the calibration period and from 0.71 to 0.84 in the validation period (Table 2). The NSE values were ≥0.5 for most of the calibrated stations, and ranged from 0.51 to 0.83 in the calibration period and from 0.56 to 0.84 in the validation period. The model performance is similar to other published work for this region [22,24,28]. The tendency of simulated versus observed monthly streamflow for the simulation period at three stations (Jordan, Clinton, Valley City, which are located in upstream, midstream and downstream of UMRB, respectively) shows that the model tracked the observed flow generally well (Figure 2a–c). The peak flows and low flows both were fitted well with the observed data throughout the time period.

**Figure A1:**
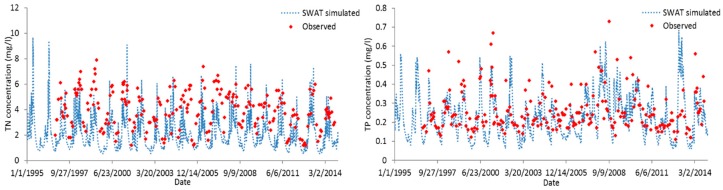
Daily simulated versus observed sediment, *TN*, and *TP* concentrations at outlet station Grafton (#05587450) in the UMRB.

The model calibration and validation of sediment and nutrient loads were satisfactory for most calibrated stations. The r^2^ (NSE) ranged from 0.54 (0.5) in Hastings to 0.75 (0.75) in Valley city for sediment calibration. The r^2^ (NSE) ranged from 0.6 (0.51) in Wapello to 0.78 (0.76) in Grafton for *TP* calibration. While for TN calibration, the r^2^ (NSE) ranged from 0.61 (0.2) in Wapello to 0.79 (0.54) in Grafton. The tendency of simulated versus observed sediment load for three stations (Hastings, Clinton and Valley City) shows that the sediment loads for most months were predicted well, although the low sediment loads for some months were slightly overestimated compared with the observed data at Clinton and Valley City (Figure 2d–f). This is potentially because the SWAT simulated low flows were slightly overestimated for some corresponding months in these two stations. The *TP* loads at Grafton were well simulated throughout the time period, although the peak values for several months were underestimated (Figure 2g). The low *TN* loads compared well with the observed data, while the peak values were underestimated compared to the observed data (Figure 2h). Overall, the model simulations do not considerably deviate from the observed trend. Most observed *TN* and *TP* concentration values were within the ranges of simulated values at Grafton, indicating good predictions of *TN* and *TP* concentrations by the model (Figure A1).

**Figure 3:**
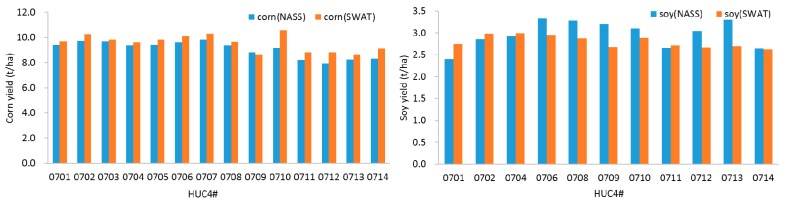
The mean annual corn and soybean of simulated (Soil and Water Assessment Tool (SWAT)) versus estimated (National Agricultural Statistics Service (NASS)) yield for each of the HUC4 subbasins in the UMRB.

The mean annual corn and soybean yields between 1995 and 2014 were overall predicted well for each HUC4 subbasin (Figure 3). The mean annual corn yield was slightly overestimated by the SWAT model for most subbasins compared to the NASS data. The percent bias (PBIAS) of corn yield ranged from −2.1 to 15.5%. The mean annual soybean yield was relatively overestimated in HUCs0701, 0702, 0704, and 0711, whereas it was underestimated in the rest of watersheds, with the PBIAS ranging from −18.5 to 14.8%. A two sample *t*-test with a confidence level of 95% (α = 0.05) was utilized to test the difference between mean annual estimated (NASS) and simulated (SWAT) yield. The result indicates that there were no significant differences (*p* > 0.05) between estimated and simulated corn yield for most subbasins. No significant differences were also observed between estimated and simulated soybean yields for HUCs0702, 0704, 0711, and 0714, with *p* > 0.05.

### 3.2. Drought Characteristic Analysis

**Figure A2:**
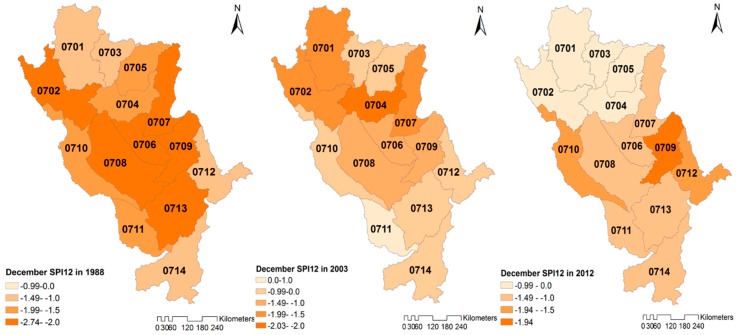
Spatial distributions of December *SPI*12 for three representative drought years (1988, 2003, and 2012).

Drought characteristics were analyzed first before evaluating their impacts on freshwater provisioning and food provisioning. Overall, the major drought years identified by three drought indices (*SPI*12, *SSWI*12, and *SSI*12) were similar, and drought commonly happened in 1987–1989, 2003, 2005, and 2012 for most HUC4 subbasins (Table 3). This successfully reconstructed the well-known historic drought events reported in the study region, such as droughts in 1988, which was the costliest drought that affected large parts of the Midwest [47]. Drought in 2005 was one of the most severe droughts in Illinois and had minor impacts on other states in the Midwest [48]. Drought in 2012 was the most recent severe and extensive drought that negatively affected agriculture in the Midwest [49]. The spatial distributions of December *SPI*12 for three representative drought years (1988, 2003, and 2012) are presented in Figure A2.

The monthly time series of *SPI*12, *SSWI*12, and *SSI*12 were highly and significantly correlated with each other with r values ≥0.7 for most subbasins (Table 4). The correlation has statistical significance, with *p* < 0.05. The maximum r value between *SPI*12 and *SSWI*12 was 0.88 in HUC0711, while the maximum r value between *SPI*12 and *SSI*12 was 0.9 in HUC0713, and the maximum r value between *SSWI*12 and *SSI*12 was 0.81 in HUC0713. Based on these results, meteorological drought index (*SPI*3) was considered for analyzing the drought persistence and frequency. Drought persistence describes drought conditions that persist from one time period to another (i.e., month-to-month, year-to-year). Drought frequency is defined as the number of drought events that occur within a study period [17,38,50,51]. In this study, the drought persistence was evaluated month-to-month and calculated as the duration of a drought event from beginning month to ending month. The tallied frequency of extreme, severe, and moderate droughts was calculated based on *SPI*3 within the 30-year period.

**Figure 4:**
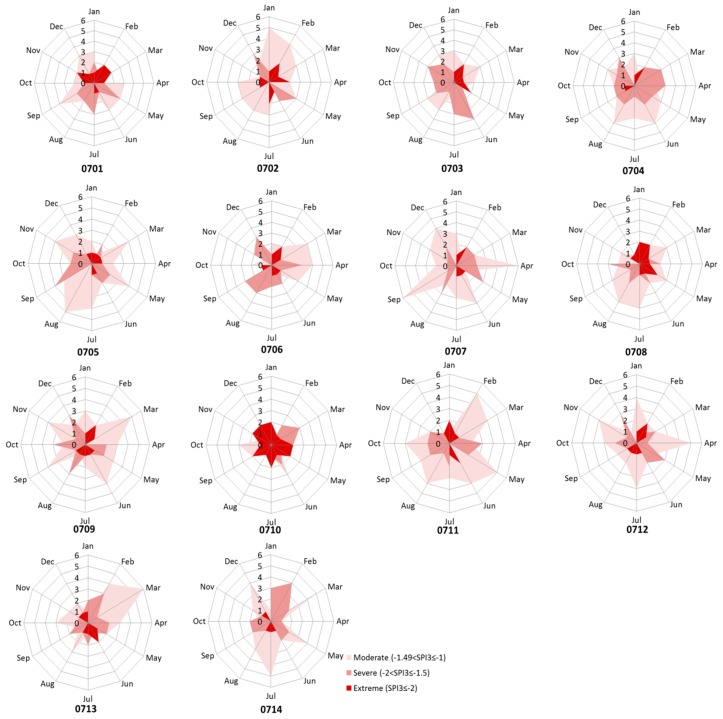
The monthly tallied frequency of drought under three categories (moderate, severe, and extreme).

**Figure A3:**
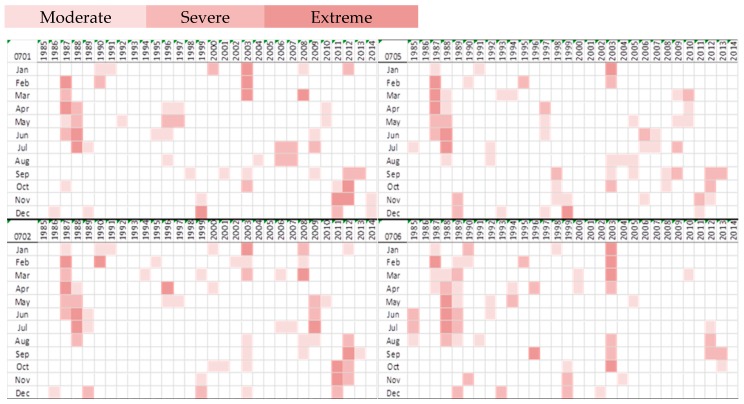

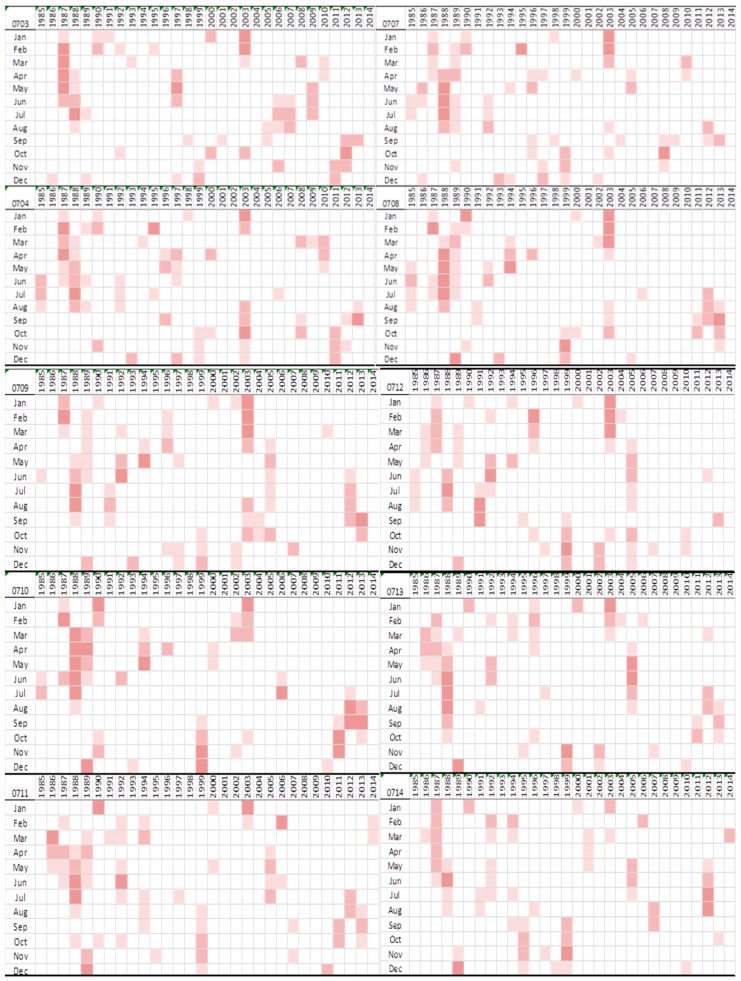
Outlook of the drought persistence for 14 HUC4 subbasins from 1985 to 2014 that was quantified by *SPI*3.

The drought persistence results (Figure A3) indicate that the most persistent drought for five upstream subbasins (HUCs0701–0705) occurred at winter-spring months of 1987 (6 months, which generally started in December of 1986 and ended in May of 1987). The highest frequency of moderate drought was counted in July–September, while the highest frequency of severe drought appeared in May and extreme drought occurred in January–February in these subbasins (Figure 4).

However, in four midstream subbasins (HUCs 0706, 0707, 0708, and 0710) and two downstream subbasins (0711 and 0713), the longest drought lasted for 4–5 months in spring and summer from March/April to August in 1988 (Figure A3). This was in agreement with several studies showing that the 1988 drought primarily occurred in spring and summer months and had a serious effect on crop yields in the Midwest of the USA [52,53]. The highest frequency of moderate drought occurred in March for three of those subbasins (0706, 0710, and 0713) and in July–September for the rest of three subbasins (0707, 0708, and 0711) (Figure 4). The severe drought occurred with highest frequency in May, August–September, while the highest frequency of extreme drought occurred in January–February for most of these subbasins.

For another two midstream subbasins (HUCs0709 and 0712), a relatively longer drought occurred in winter-spring months of 2003 which generally started in November 2002 and ended in April 2003 (Figure A3). The moderate drought occurred with the highest frequency in March and April in these two subbasins (Figure 4). The highest frequency of severe drought occurred in August, October, and December in HUC0709 while it happened in May in HUC0712. The moderate drought primarily occurred in July in HUC0714, while severe drought happened with highest frequency in February.

### 3.3. Drought Impacts on Freshwater Provisioning

#### 3.3.1. Meteorological Drought Impacts on Annual Freshwater Provisioning

**Figure 5:**
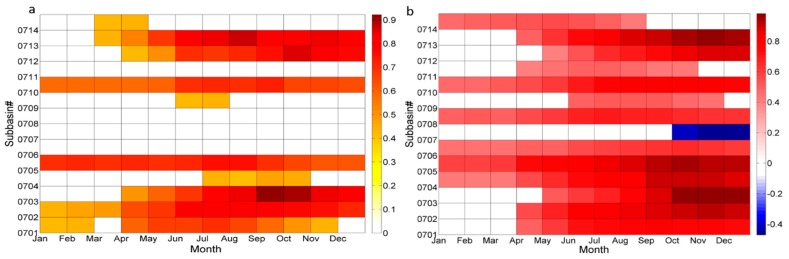
The correlation coefficients (r) between annual freshwater provisioning (*FWP*) and *SPI*12 series (**a**); as well as *SSI*12 series (**b**) for each month in 14 HUC4 subbasins.

The correlation coefficients between annual *FWP* and *SPI*12 for each subbasin indicate that the annual *FWP* was overall positively correlated with *SPI*12 for each month within the 30-year period (Figure 5a). Considering that the r value between −0.3 and 0.3 represents a weak correlation, we only included the correlation higher than |0.3|. Comparing the r values between *FWP* and *SPI*12 in different months, higher correlations were observed in the last 6 months of the year than that in the first 6 months for seven subbasins (HUCs0701, 0702, 0703, 0705, 0710, 0712, and 0713). The correlation had statistical significance (*p* < 0.05). Specifically, in HUC0705 and HUC0710, the correlation coefficient peaked in July and September with values of 0.76 and 0.72, respectively (*p* < 0.05). In HUCs0702, 0703, 0712, and 0713, the r value was overall above 0.7 for the last 6 months of the year, with *p* < 0.05 indicating a significant correlation. The maximum r values were 0.83, 0.92, 0.85, and 0.86, which occurred in August, September, October, and August, respectively.

Based on the above results, we mainly focused on correlations for the last 6 months of each year to make a spatial comparison for different subbasins. Spatially, the annual *FWP* was highly and significantly correlated with *SPI*12 in HUCs0702, 0703, 0712, and 0713 with r > 0.7 and *p* < 0.05. In HUC0705 and HUC0710, the correlation between annual *FWP* and *SPI*12 was moderate and significant in December (r = 0.63 and 0.64, *p* < 0.05, respectively). However, in the headwater subbasin HUC0701 and upstream subbasin HUC0704, the correlation was weak, with r < 0.5 in the last 6 months. For other midstream subbasins (HUCs0706, 0707, 0708, 0709) and two downstream subbasins (HUC0711 and HUC0714), there was no correlation between annual *FWP* and *SPI*12, with r ˂ 0.3 and *p* > 0.05. This indicates that the annual freshwater provisioning in these subbasins is less affected by precipitation deficits and/or surplus in the second half of the year.

#### 3.3.2. Hydrological Drought Impacts on Annual Freshwater Provisioning

Streamflow and water quality have larger impacts on *FWP* compared to precipitation. Thus, the correlation coefficients between annual *FWP* and *SSI*12 were calculated for analyzing the hydrological drought impacts on *FWP*. The correlations between annual *FWP* and *SSI*12 generally show a similar result to *SPI*12, with maximum r observed during the last 6 months of the year (July–December) (Figure 5b).

Spatially, the annual *FWP* was better correlated with *SSI*12 than *SPI*12 in almost all subbasins. For those subbasins located on tributary rivers (HUCs0702, 0703, 0705, 0710, 0712, and 0713), the annual *FWP* was highly correlated with *SSI*12 in the last 6 months of the year, with r > 0.7. The correlation had statistical significance, with *p* < 0.05. In HUC0701 and HUC0704, the correlation between annual *FWP* and *SSI*12 was relatively high and significant in the last 6 months, with r > 0.7 and *p* < 0.05, while the correlation between annual *FWP* and *SPI*12 was relatively weak. In HUC0706 and HUC0708, the annual *FWP* was moderately and insignificantly correlated with *SSI*12 (*p* > 0.05), while it was weakly correlated with *SPI*12. In HUCs0709, 0711, and 0714, the correlations were also a bit improved compared to that between annual *FWP* and *SPI*12 although it was still weak, with r ˂ 0.5 and *p* > 0.05. These results indicate that the annual *FWP* is relatively more affected by hydrological drought than meteorological drought.

However, the correlations between annual *FWP* and *SSI*12 as well as *SPI*12 were generally weak in HUCs0707, 0709, 0711, and 0714. Particularly, in HUC0707, the correlations were relatively weak and negative (r ≈ −0.4) in the last three months (October–December), indicating that the annual *FWP* may be improved under dry conditions and may get worse under wet conditions in this subbasin.

#### 3.3.3. Meteorological Drought Impacts on Seasonal Freshwater Provisioning

Generally, a large number of droughts occur with duration less than one year and therefore, it is necessary to analyze the short-term drought effects on freshwater provisioning on a seasonal scale.

**Figure 6:**
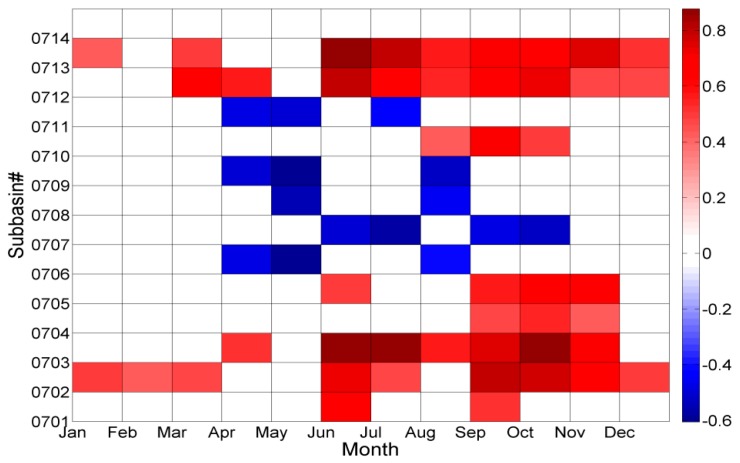
The correlation coefficients (r) between seasonal *FWP* and *SPI*3 per month in 14 HUC4 subbasins.

The correlation coefficients between seasonal *FWP* and *SPI*3 per month for each subbasin indicate that the *FWP* was highly correlated with *SPI*3 in summer and autumn for most of subbasins (Figure 6, correlations ≥|0.3| were just included in the figure). Specifically, the r values peaked in June, with 0.88, 0.8, and 0.88 in HUCs0703, 0712, and 0713, respectively. The correlation had statistical significance (*p* < 0.05). The maximum r values occurred in September, with 0.8 and 0.7 in HUC0702 and HUC0710, respectively. These results indicate that freshwater provisioning is more affected by the precipitation deficits and/or surplus in summer and autumn.

However, a moderate and negative correlation was observed between *FWP* and *SPI*3 in spring and summer (April–August) for HUCs0706, 0707, 0708, 0709, and 0711. The correlation had statistical significance, with *p* < 0.05. The minimum r values occurred in May for HUCs0706, 0708, 0709, and 0711, with values of −0.60, −0.54, −0.61, and −0.51, respectively. While the minimum r value appeared in July in HUC0707 with a value of −0.56. These results indicate that the freshwater provisioning is negatively affected by the precipitation deficits and/or surplus during spring and summer in these five subbasins.

### 3.4. Meteorological Drought and Agricultural Drought Impacts on Food Provisioning

**Figure 7:**
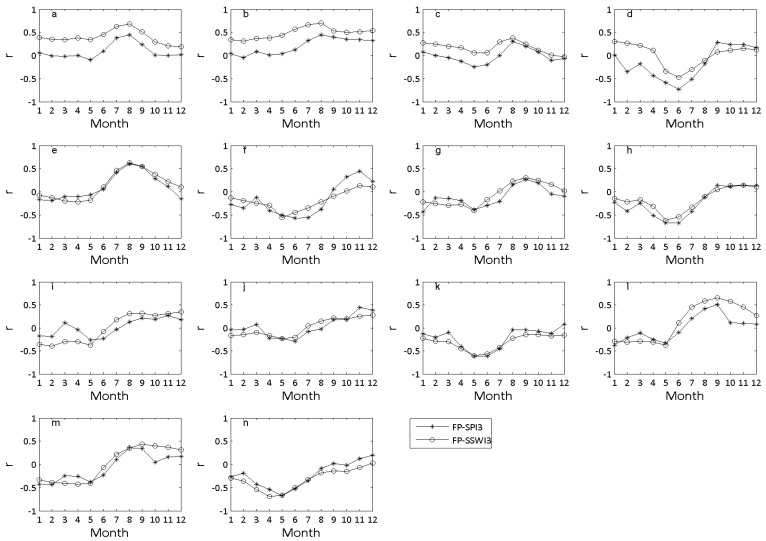
The correlation coefficients (r) between annual *FP* and *SPI*3 as well as *SSWI* at a 3-month scale (*SSWI*3) for each month in 14 HUC4 subbasins: 0701 (**a**); 0702 (**b**); 0703 (**c**); 0704 (**d**); 0705 (**e**); 0706 (**f**); 0707 (**g**); 0708 (**h**); 0709 (**i**); 0710 (**j**); 0711 (**k**); 0712 (**l**); 0713 (**m**); 0714 (**n**).

The correlation coefficients between annual *FP* and *SPI*3 show that the annual *FP* was overall positively correlated with *SPI*3 during the last 6 months of the calendar year for most subbasins (Figure 7). The correlation coefficients between July and September were relatively higher than other months for HUCs0701, 0702, 0703, 0705, 0712, and 0713. The correlation has statistical significance, with *p* < 0.05. The maximum r value in August was 0.61 for HUC0705. Similarly, the correlation coefficients between *FP* and *SSWI*3 were also higher from July to September than other months in these subbasins. The maximum r value in August was 0.71 for HUC0702 (*p* < 0.05). The results suggest that the annual food provisioning is more affected by the precipitation and soil water deficits and/or surplus during summer and early autumn in these subbasins.

Compared to summer and autumn, very weak correlations between annual *FP* and *SPI*3 as well as *SSWI*3 were observed during winter months (January–February, December) (Figure 7). The r values of annual *FP* with *SPI*3 were between −0.3 and 0.3 during this period for most of the subbasins. No statistical significance was observed, with *p* > 0.05. The r values of annual *FP* with *SSWI*3 also ranged from −0.3 to 0.3 during winter for most of subbasins, with *p* > 0.05. These results indicate that the annual food provisioning is less affected by the precipitation and soil water deficits in winter. However, a moderate and negative correlation between annual *FP* and *SPI*3 as well as *SSWI*3 was observed in spring for eight subbasins (HUCs0704, 0706–0708, 0711–0714). The correlation has statistical significance, with *p* < 0.05. The minimum r value between annual *FP* and *SPI*3 in May was −0.68 in HUC0708, whereas the minimum r value between annual *FP* and *SSWI*3 in May was −0.66 in HUC0714.

A similar trend was observed for all subbasins when the correlations between annual *FP* and *SPI*3 as well as *SSWI*3 were compared (Figure 7). The r values between *FP* and *SSWI*3 were consistently higher than that between *FP* and *SPI*3 for each month for HUCs0701–0704 and HUC0712. For the rest of the subbasins, the r values between *FP* and *SSWI*3 were slightly greater than that between *FP* and *SPI*3 during summer and early autumn months from June to September. These results suggest that the annual food provisioning was relatively more affected by agricultural drought than meteorological drought during crop reproductive stages.

## 4. Discussion

### 4.1. Drought Impacts on Annual Freshwater Provisioning

The results of this study indicate that annual *FWP* was overall positively correlated with *SPI*12 for each month within the 30-year period. Higher correlations were observed in the last 6 months of the year than that in the first 6 months, with *p* < 0.05. The annual *FWP* represents freshwater provisioning quantities through the total 12 months of each year. The *SPI*12 in the second half of the year such as December can better reflect the precipitation pattern of the entire year [54]. Thus, it is not unexpected that relatively high correlations between annual *FWP* and *SPI*12 appeared in the last 6 months of the year.

The result also indicates that the annual *FWP* is relatively more affected by hydrological drought than meteorological drought. The correlations between annual *FWP* and *SSI*12 were significantly improved comparing with *SPI*12, particularly in HUCs0701, 0704, 0706, 0708, 0709, 0711, and 0714. Considering the watershed characteristics of these subbasins, they are all located on the main rivers except HUC0701 and HUC0709 (Figure 1). The *FWP* in these mainstream subbasins would be influenced by inflows and nutrient inputs from upstream subbasins. The streamflow deficits at outlet of each subbasin would directly affect the *FWP*, while precipitation is an indirect factor affecting *FWP*. Thus, the annual *FWP* generally showed a better correlation with *SSI*12 than *SPI*12, which indicates a greater importance of hydrological drought implications on freshwater provisioning.

**Figure 8:**
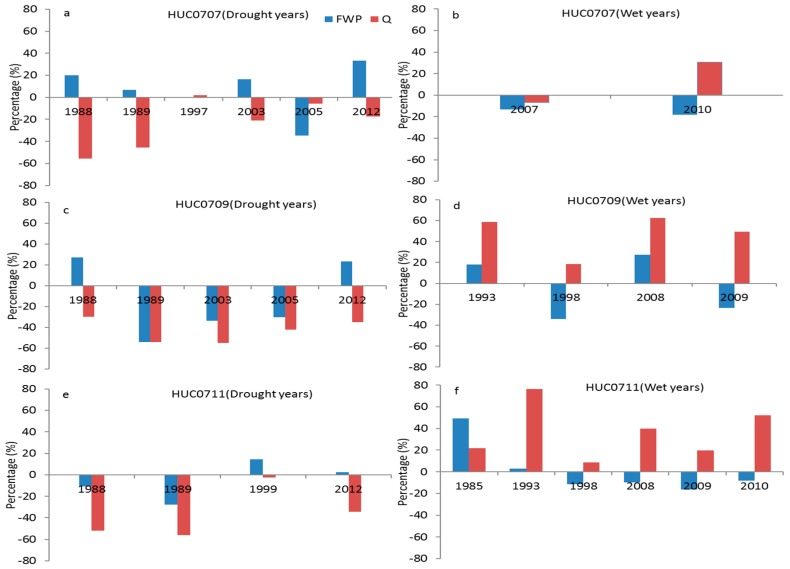
The percentage changes of annual *FWP* and total flow (Q) at drought years (**a**,**c**,**e**) and wet years (**b**,**d**,**f**) based on the 30-year mean value for three subbasins (HUC0707, HUC0709, and HUC0711).

A generally weak correlation between annual FWP and *SSI*12 as well as *SPI*12 was observed in HUCs0707, 0709, 0711, and 0714. As the *FWP* is a function of flow and water quality, the weak correlations between annual *FWP* and two drought indices in these subbasins may be caused by many factors, such as watershed slope, type of nutrient sources and routing, etc. In order to further explain the weak correlations in these subbasins, the percentage changes of *FWP* and their determinative factors in drought years (*SPI* < −1) and wet years (*SPI* > 1) based on a 30-year mean value were calculated. The annual *FWP* in HUC0707 increased by a range of 0.5 to 33.5% in most drought years (Figure 8a), while it decreased by −13.1% and −18.1% in two wet years (Figure 8b). The annual *FWP* in HUC0709 increased by 27.1% and 23.2% in 1988 and 2012, respectively (Figure 8c), whereas it decreased by −34% and −23.3% in 1998 and 2009, respectively (Figure 8d). In HUC0711, the annual *FWP* increased by 14.6% and 2.5% in 1999 and 2012, respectively (Figure 8e), while it reduced by a range of −8.3 to −11.4% in four wet years (Figure 8f). The increased *FWP* in some drought years as well as decreased *FWP* in wet years in these subbasins may result in weak correlations between annual *FWP* and two drought indices.

**Figure 9:**
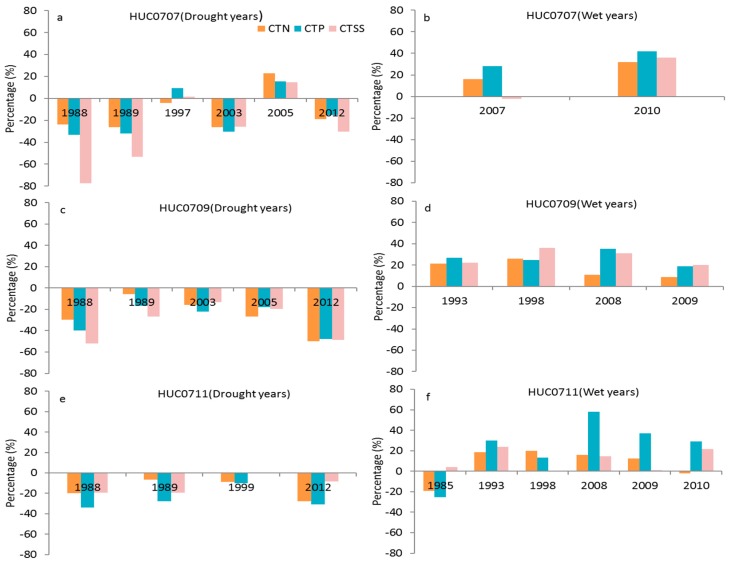
The percentage changes of *TN*, *TP* and *TSS* concentrations (C_TN_, C_TP_, C_TSS_) in drought years (**a**,**c**,**e**) and wet years (**b**,**d**,**f**) based on the 30-year mean value for three subbasins (HUC0707, HUC0709, and HUC0711).

The *FWP* is affected by both flow and water quality, with water quality having a greater influence [43]. As shown in Figure 8a–f, the total flow decreased in most drought years and increased in most wet years in each subbasin. The concentrations of *TN*, *TP*, and *TSS* also generally decreased in most drought years, while they increased in most wet years (Figure 9a–f). This is consistent with several studies that have shown that nutrient and sediment often decrease during droughts in rivers and streams, primarily because of the reduced loadings from agricultural non-point sources and the increased influence of internal processes such as biological uptake of nutrients, denitrification, and sediment settling [7,8]. Thus, the water quality conditions of these subbasins will be potentially improved in drought years while reduced in wet years. Although the streamflow increased in wet years, the reduced water quality condition in the same period may result in a reduction of *FWP*. On the other hand, the decreased streamflow in drought years may not reduce the *FWP* because of the improved water quality condition during the same period [7,8,43]. As a result, the annual *FWP* displayed a weak correlation with both drought indices (*SPI*12 and *SSI*12) in HUCs0707, 0709, 0711, and 0714, indicating that the annual freshwater provisioning is less affected by precipitation or streamflow deficits and/or surplus in these subbasins.

### 4.2. Meteorological Drought Impacts on Seasonal Freshwater Provisioning

The results from this study indicate that seasonal freshwater provisioning is more affected by the precipitation deficits and/or surpluses in summer and autumn in most of subbasins. As discussed in Section 3.2, summer and early autumn (July–September) are the seasons where the highest frequency of moderate drought occurred. The river flow in summer is sensitive to precipitation and evapotranspiration (ET), with less availability during the crop growing season between June and August and the greatest seasonal water demand approximately occurring in mid-July [13,55]. The precipitation and streamflow deficits during summer and autumn would highly affect freshwater provisioning.

However, the seasonal freshwater provisioning is negatively affected by the precipitation deficits and/or surpluses in spring and summer for HUCs0706, 0707, 0708, 0709, and 0711. As discussed in Section 3.3.3, water quality has a greater influence on *FWP*. The sediment and nutrient loads and concentrations usually increase during high rainfall season and reduce during dry periods [7,8]. A highly positive and significant correlation between *SPI*3 and monthly sediment load as well as nutrient loads has already been observed in these five subbasins during spring and summer, with r values ≥ 0.7 and *p* < 0.05. As most of these subbasins have agriculture as the dominant land use/cover (Table 1), great amounts of sediment and nutrients would be transported from landscapes to streams and rivers via surface and subsurface pathways in spring and early summer [19,56,57]. For example, nitrate leaching from soil during high rainfall season would increase the concentration of nitrate and subsequently may decline the water quality conditions in the rivers. A positive correlation between nutrient concentrations and *SPI*3 during spring and summer indicates relatively good water quality conditions in dry periods while declined conditions in wet periods. As a result, freshwater provisioning would be reduced because of declined water quality conditions in wet periods. The correlation between *FWP* and *SPI*3 became negative during spring and summer.

### 4.3. Meteorological Drought and Agricultural Drought Impacts on Food Provisioning

The results suggest that the annual food provisioning is more affected by the precipitation and soil water deficits and/or surpluses during summer and early autumn in some subbasins (HUCs0701, 0702, 0703, 0705, 0712, and 0713), while it is less affected by the precipitation and soil water deficits in winter. These results are consistent with previous studies showing peak correlations between crop yield and drought indices or drought stress intensity during crop growth stages from July to September [15,18,58,59]. For example, as stated in Johnson [58], the peak r value between corn yields and Normalized Difference Vegetation Index (NDVI) was 0.73 during the first week of August, while it was 0.7 between soybean yield and NDVI a week later in August. Kogan et al. [59] concluded that a strong correlation of corn production with vegetation condition index (VCI) occurred between August and mid-September, with r values of 0.78–0.88. The r value between corn yield and drought stress intensity at reproductive stage was −0.37, as reported in Wang et al. [18]. Corn and soybean are major crops planted in the Midwest. The months from July to September are the critically reproductive periods (from head or silk to physiological maturity) for these two crops [13,17,59,60]. Critical growth phases during this period make the crop more susceptible to weather conditions. Pollen viability and silk receptivity can be reduced under dry conditions with high temperature stress and low moisture, leading to a poor seed set and reduced crop yield. As shown in Section 3.2, the highest frequency of moderate drought was observed in summer and early autumn months (July to September). The highest soil moisture for plant water uptake would be required for corn and soybean during these growth stages. Thus, relatively higher and significant correlations between food provisioning and *SPI*3 as well as *SSWI*3 were consistently observed during summer and early autumn. As the crop calendar of corn and soybean is from April to November in the Midwest USA [59,60], the precipitation or soil moisture conditions in winter may not play as important role as other months in improving crop production.

However, a moderate and negative correlation between annual *FP* and *SPI*3 as well as *SSWI*3 was observed in spring for eight subbasins (HUCs0704, 0706–0708, 0711–0714). Considering watershed characteristic of these subbasins, most of them are cropland dominant watersheds and located on the mainstreams (Table 1). The moderate and negative correlations between *FP* and two drought indices indicated crop production is relatively less affected by drought during the planting period (April–June). Lobell et al. [16] noted that crop yields are becoming less sensitive to drought during early growing stages, probably because expanded low or no-till systems may increase the soil moisture under drought conditions. Johnson [58] reported a very weak correlation between precipitation and crop yields during the planting period and concluded that the soil moisture may build up over a longer time period with enough depth for plants to reach and uptake, even though less rainfall occurred during this period [16,58]. The water deficit during spring is important, but excess precipitation and soil moisture will be harmful to the food provisioning. In other words, excess water stress may negatively affect crop yield [18]. Spring and early summer represent the rainfall season in the Midwest USA. The high frequency of quick storms or long durations of precipitation events would increase the risk of flooding and may result in excess water in croplands, which can potentially prevent crop growth and reduce food provisioning.

The results from this study also indicate that annual food provisioning was relatively more affected by agricultural drought than meteorological drought during crop reproductive stages. Since the amount of soil moisture in the root zone is a more important factor for crop growth than precipitation [15,61], the soil moisture deficits during various crop growth stages (particularly reproductive stages) would have a substantial effect on crop production. The *SSWI*3 directly reflects the soil moisture conditions at different stages of crop growth, while the *SPI*3 represents short-term precipitation patterns which indirectly affect crop production. Therefore, a better correlation between annual *FP* with *SSWI* was observed for most subbasins during crop reproductive stages, indicating that agricultural drought was more important for food provisioning.

## 5. Conclusions

A greater importance of hydrological drought than meteorological drought for freshwater provisioning was evident for most subbasins. The seasonal *FWP* was highly affected by the precipitation deficits and/or surpluses in summer and autumn for most of the subbasins. For some agriculturally dominant subbasins, the *FWP* was moderately and negatively affected by precipitation deficits and/or surpluses during spring and summer primarily because of the improved water quality conditions during dry periods and declined water quality conditions during wet periods. Food provisioning was more affected by the precipitation and soil water deficits during summer and early autumn (July–September), with relatively less effect observed in winter. A greater importance of agricultural drought effects on food provisioning was evident for most subbasins during crop reproductive stages. For some mainstream subbasins, the food provisioning service was relatively less affected by drought during spring, as shown by moderate and negative correlations between annual *FP* and two drought indices (*SPI*3 and *SSWI*3).

This study is based on past observed droughts that impacted freshwater provisioning and food provisioning services under baseline climate conditions. Future work should consider multiple climate change and variability scenarios relating to droughts and floods, and investigate the responses of freshwater and food provisioning services under future climate changes. Such results would be helpful for land managers to make adaptive strategies to ease the extreme climate impacts and protect ecosystem services in the UMRB.

## Figures and Tables

**Table 1:** A summary of 14 HUC4 subbasins in the UMRB along with information on main land use types for each HUCs.

#HUC4	Subbasin Name	States	Drainage Area (10^3^ km^2^)	Slope (%)	Cropland (%)	Pasture (%)	Forest (%)
0701	Mississippi Headwaters	MN	96.1	2.4	14	3	42
0702	Minnesota	IA, MN	44.1	2.3	70	9	3
0703	St. Croix	WI, MN	20	3.3	3	10	60
0704	Upper Mississippi-Black-Root	MN, WI	168.7	9.6	28	20	37
0705	Chippewa	WI	24.7	4.7	7	7	57
0706	Upper Mississippi-Maquoketa-Plum	IA, WI	221.8	9.3	33	31	25
0707	Wisconsin	WI	30.9	7.3	9	13	51
0708	Upper Mississippi-Iowa-Skunk-Wapsipinicon	IA	309.4	3.6	66	17	6
0709	Rock	IL, WI	28.2	3.3	50	20	11
0710	Des Moines	IA, MN	37.4	3.8	64	15	10
0711	Upper Mississippi-Salt	IL, MO, IA	447.6	4.6	32	32	27
0712	Upper Illinois	IL, IN	28.3	1.7	50	9	10
0713	Lower Illinois	IL	74.7	3.0	65	9	15
0714	Upper Mississippi-Kaskaskia-Meramec	IL, MO	491.9	4.6	27	19	42

**Table 2:** Monthly streamflow calibrated stations and statistical results in the UMRB. USGS, US Geological Survey; NSE, Nash-Sutcliffe efficiency.

HUC4 Subbasin	Station Name	USGS Station#	Calibration		Validation	
			r^2^	NSE	r^2^	NSE
Mississippi Headwaters	St. Paul	05331000	0.75	0.59	0.8	0.7
Minnesota	Jordan	05330000	0.85	0.83	0.81	0.72
St. Croix	St. Croix	05340500	0.66	0.53	0.71	0.19
Chippewa	Durand	05369500	0.7	0.65	0.76	0.73
Upper Mississippi-Maquoketa-Plum	McGregor	05389500	0.73	0.64	0.79	0.56
Wisconsin	Muscoda	05407000	0.7	0.65	0.75	0.62
Upper Mississippi-Iowa-Skunk-Wapsipinicon	Clinton	05420500	0.7	0.6	0.79	0.58
Upper Mississippi-Iowa-Skunk-Wapsipinicon	Augusta	05474000	0.76	0.64	0.83	0.8
Upper Mississippi-Iowa-Skunk-Wapsipinicon	Wapello	05465500	0.74	0.68	0.82	0.77
Rock	Joslin	05446500	0.85	0.51	0.81	0.4
Des Moines	Keosauqua	05490500	0.75	0.75	0.84	0.84
Upper Mississippi-Salt	Grafton	05587455	0.81	0.72	0.83	0.74
Lower Illinois	Valley City	05586100	0.84	0.77	0.79	0.61

**Table 3:** Major drought years for each of the HUC4 subbasins identified using the 12-month standardized precipitation index (*SPI12*), the 12-month standardized streamflow index (*SSI12*), and the 12-month standardized soil water content index (*SSWI12*) for the period 1985–2014.

HUC4 Subbasins	0701	0702	0703	0704	0705	0706	0707	0708	0709	0710	0711	0712	0713	0714
Drought years	1987	1987	1987	1988	1987	1988	1988	1988	1988	1988	1988	1988	1987	1988
	1988	1988	1988	1989	1988	1989	1989	1989	1989	1989	1989	1989	1988	1989
	1989	1989	1989	2003	1989	2003	1997	2003	2003	2012	1999	2002	1989	1991
	1992	2003	1992		2006	2012	2003	2012	2005		2012	2005	2005	1992
	2003		1997		2009		2005		2012			2012	2012	2007
	2006		2006				2012							2012
			2009											

**Table 4:** Correlation coefficients between *SPI*, *SSWI*, and *SSI* for each subbasins within a 30-year period.

Correlation Coefficient (r)	0701	0702	0703	0704	0705	0706	0707	0708	0709	0710	0711	0712	0713	0714
*SPI*12 vs. *SSWI*12	0.54 *	0.70 *	0.48 *	0.61 *	0.58 *	0.67 *	0.47 *	0.78 *	0.78 *	0.68 *	0.88 *	0.78 *	0.86 *	0.73 *
*SPI*12 vs. *SSI*12	0.72 *	0.87 *	0.87 *	0.58 *	0.83 *	0.60 *	0.61 *	0.73 *	0.81 *	0.90 *	0.76 *	0.87 *	0.90 *	0.58 *
*SSWI*12 vs. *SSI*12	0.52 *	0.75 *	0.47 *	0.55 *	0.63 *	0.60 *	0.51 *	0.70 *	0.76 *	0.73 *	0.78 *	0.75 *	0.81 *	0.62 *

* Denotes significant correlations (*p* < 0.05) between *SPI12*, *SSI12*, and *SSWI12*.
